# Tumeur d'Abrikossoff juxta axillaire: une localisation rare d'une tumeur très rare, à propos d'un cas

**DOI:** 10.11604/pamj.2015.21.164.6622

**Published:** 2015-06-26

**Authors:** Sarah Amourak, Wael Bouzoubaa, Sofia Jayi, Fatimazahra Fdili Alaoui, Hikmat Chaara, Moulay Abdelilah Melhouf

**Affiliations:** 1 Service de Gynécologie Obstétrique 2, Université sidi Mohammed Benabdellah, CHU Hassan II de Fès, Maroc

**Keywords:** Tumeur d´Abrikossoff, tumeur à cellules granuleuses, origine neurogène, immunohistochimie, Abrikossoff tumor, Granular cell tumor, neurogenic, immunohistochemistry

## Abstract

Décrite pour la première fois en 1926 par Abrikossoff, les tumeurs à cellules granuleuses (TCG) sont bénignes et uniques dans la grande majorité des cas. Les principales localisations sont la cavité orale, puis les tissus sous-cutanés de la tête et du cou et les seins. La pathogenèse a été longtemps débattue, après avoir proposé initialement une origine musculaire striée les études récentes sont en faveur d'une origine neurogène schwannienne confirmées par une étude immunohistochimique. Le traitement de la tumeur à cellules granuleuses est un traitement chirurgical, il permet un diagnostic de certitude par l'examen anatomo-pathologique de la pièce d'exérèse qui doit rechercher les limites d'exérèse et la présence de critères de malignité. Leur évolution est souvent favorable si la résection chirurgicale est complète. Nous rapportons ici le cas d'une tumeur d'Abrikossoff à localisation juxta axillaire et nous profitons de faire une revue de la littérature afin de mettre le point sur cette entité rare.

## Introduction

Décrite pour la première fois en 1926 par Abrikossoff, les tumeurs à cellules granuleuses (TCG) sont bénignes et uniques dans la grande majorité des cas. Les principales localisations sont la cavité orale, puis les tissus sous-cutanés de la tête et du cou et les seins.

## Méthodes

A travers notre cas et une revue de la littérature, nous essayerons de préciser les caractéristiques diagnostiques, thérapeutiques, pronostiques de cette entité rare.

## Résultats

Patiente âgée de 47ans, ayant comme antécédent une hystérectomie pour un utérus polymyomateux, qui présente depuis 3mois une tumeur juxta-axillaire droite faisant 2cm, mobile, indolore et sans signes inflammatoires en regard, l'examen des seins est sans particularité. Elle a bénéficié d'une échographie mammaire+ mammographie: seins mammographiquement et échographiquement normaux, absence d'adénopathies axillaires. Présence d'une masse juxta-axillaire droite de 20mm avec quelques rares vaisseaux. La patiente a bénéficié d'une exérèse de la tumeur dont le résultat anatomopathologique est en faveur d'une tumeur à cellule granuleuse (Tumeur d'Abrikossoff).

## Discussion

La tumeur à cellule granuleuse d'Abrikossoff est une tumeur habituellement bénigne. C'est une tumeur rare, de siège ubiquitaire avec prédilection pour la localisation linguale [1]. Elle se développe entre 20 et 60 ans et serait plus fréquente chez la femme. La pathogenèse a été longtemps débattue, après avoir proposé initialement une origine musculaire striée les études récentes sont en faveur d´une origine neurogène schwannienne confirmées par une étude immunohistochimique [2]. Histologiquement, la tumeur est constituée par des plages irrégulières ou de petits amas polyédriques ou ovalaires de cellules spécifiques, à cytoplasme riche en fines granulations et faiblement éosinophile. Les limites cytoplasmiques sont bien marquées. Le noyau est de petite taille, plutôt central et ovalaire, avec une chromatine généralement homogène, rarement hyperchromatique. Il ne présente pas de mitose. Les amas cellulaires sont entourés par des travées conjonctives riches en collagènes et en réticuline, et les cellules granuleuses peuvent présenter des intrications avec des éléments musculaires ou nerveux du voisinage. L´épithélium de recouvrement des tumeurs superficielles est très fréquemment le siège d´une hyperplasie pseudo-épithéliomateuse, Cette hyperplasie résulterait soit d´une irritation locale soit d´une sécrétion de facteurs perturbants par les cellules granuleuses. Il pourrait s´agir d´enzymes lysosomiales accumulées au sein de ces cellules granuleuses et libérées lors de nécroses cellulaires survenant pendant la croissance tumorale. Des infiltrations de fibres musculaires striées sont aussi observées entre les cellules granuleuses Il existe des formes malignes [3] dans moins de 2% des cas, suspectées cliniquement par une taille de plus de 4 cm, des plages nécrotiques et/ou hémorragiques et une croissance rapide. Histologiquement, on retrouve de nombreuses atypies cellulaires, des cellules tumorales en mitose et présentant des nécroses. L'immunohistochimie retrouve une forte proportion de noyaux positifs au marqueur de prolifération Ki67 [4]. Les formes malignes nécessitent un bilan radiologique à la recherche de localisations secondaires lymphatiques ou systémiques (poumon, foie, os). Le diagnostic de certitude restera histologique. Le traitement de la tumeur à cellules granuleuses est un traitement chirurgical, il permet un diagnostic de certitude par l'examen anatomo-pathologique de la pièce d'exérèse qui doit rechercher les limites d'exérèse et la présence de critères de malignité [1]. La patiente est considérée guéri après cette exérèse locale, sauf dans les cas suivants: Tumeur avec multiples lésions,Tumeur mesurant plus de 5 cm, Tumeur avec croissance rapide, Tumeur avec récurrence locale. Ces tumeurs nécessitent une surveillance ultérieure étroite car elles présentent un risque de malignité et de récidive. Leur évolution est souvent favorable si la résection chirurgicale est complète. Néanmoins, en cas de résection tumorale incomplète, la persistance de la tumeur ou sa récidive tumorale est inconstante [5].

## Conclusion

La tumeur d'Abrikossoff est une tumeur bénigne rare. Son diagnostic de certitude est exclusivement histologique et immunohistochimique. Une exérèse chirurgicale complète constitue le traitement de choix.
